# “Does this dental mob do eyes too?”: perceptions and attitudes toward dental services among Aboriginal Australian adults living in remote Kimberley communities

**DOI:** 10.1186/s12903-021-02003-2

**Published:** 2021-12-25

**Authors:** Jilen Patel, Angela Durey, Steven Naoum, Estie Kruger, Linda Slack-Smith

**Affiliations:** 1 School of Population and Global Health, The University of Western Australia, Perth, Australia; 2 UWA Dental School, The University of Western Australia, Perth, Australia; 3 School of Human Sciences, The University of Western Australia, Perth, Australia

**Keywords:** Aboriginal, Dental, Oral health, Perceptions, Service delivery, Treatment

## Abstract

**Background:**

Australian Aboriginal and Torres Strait Islander people continue to experience significant disparities in oral health and there remains an urgent need to improve services to rural and remote communities. Quantitative research has typically been used to highlight the disease burden and severity experienced by those living in remote communities, but this data does little to explore the lived reality and psychosocial nuances that impact on care. The Kimberley region of Western Australia is home to over 150 Aboriginal communities spread out across 400,000 square kilometres. The success and sustainability of oral health services to these remote communities relies on respect and reciprocity achieved through shared knowledge, decision making and involvement of Aboriginal people in discussions around oral health services and their delivery. This, study aimed to investigate the perceptions and attitudes toward dental services among Aboriginal Australian families living in remote Kimberley communities.

**Methods:**

Semi-structured interviews and yarning circles were carried out following purposive sampling of Aboriginal adults living in the East Kimberley region of Western Australia. Interviews were recorded, transcribed, and analysed guided by a constructivist grounded theory approach.

**Results:**

In total, 80 community members participated in the yarning process. Enablers to care included: promotion of existing services, integration with primary health services, using mobile dental services and volunteers to extend care. Barriers to care included transportation, cost of treatment, the complexity of appointment systems and shame associated with health-seeking behaviours.

**Conclusions:**

Reassessing the prevailing operative model of dental care to remote Aboriginal communities is warranted to better address the overwhelming structural barriers that impact on oral health. Integration with existing primary health services and schools, the use of mobile units to extend care and increasing community engagement through clinical yarning are recommended in improving the current state of dental services to communities in the Kimberley.

## Introduction

Oral health interventions have had limited success in Indigenous communities and all too often have failed to create sustainable improvements in oral health outcomes [1]. Respect and reciprocity achieved through shared knowledge and involving Aboriginal people in decisions about their health are critical factors towards success [2]. This is in contrast to top-down approaches typically applied through a colonial lens which without adequate consultation, fail to create lasting change [3]. Jamieson and co-workers note the poor quality of existing data in addition to a scarcity of qualitative data investigating the attitudes and perceptions of oral health in Aboriginal communities and note the usefulness of such research in directing culturally appropriate initiatives [4]. More recently Tynan and colleagues used a phenomenological approach to facilitate a deeper contextual understanding around oral health attitudes in Aboriginal communities [5]. The use of such qualitative methods has highlighted the importance of this technique in identifying local barriers to care, community priorities and strategies to ameliorate services in the future [5, 6]. This paper focuses on the Kimberley region of Western Australia, noting that previous literature has suggested that Aboriginal Australians living in the Kimberley are *the most disadvantaged group within the most disadvantaged population in Australia* and continue to face significant disparities in oral health [7] ^(p2)^. Providing adequate access to timely dental care to many areas of the Kimberley is both a challenge and a priority and there remains an urgent need to develop alternative strategies to improve the oral health of remote Aboriginal communities in this region [8, 9].

A better understanding of the complex and challenging lives of Aboriginal people living in the Kimberley is required, where basic concerns about personal safety and having enough money to provide food and housing may take precedence over health behaviours such as accessing dental care and maintaining adequate oral hygiene [10, 11]. In order to better inform future dental services to remote communities, this study aimed to investigate the perceptions and attitudes among Aboriginal Australian families, living in the Kimberley, towards dental services.

### Methodology

This qualitative study was conducted following engagement with remote Kimberley communities that occurred as part of Kimberley Dental Team (KDT) visits to the region between 2010 and 2015. The KDT is a not-for-profit, non-government volunteer-led organisation that has been providing dental services to remote Kimberley communities by invitation. As part of these visits, councillors in the East Kimberley, school teachers, Aboriginal liaison officers, hospital staff and members of the Aboriginal community, were consulted on the accessibility of dental care and the nature of services provided to communities. In addition, discussions arising from partnership with the Aboriginal Medical Service and local Aboriginal Controlled Community Health Services in the East Kimberley informed the design and purpose of this study. This study was formally commenced in 2015 with ethics approval being obtained from UWA Human Research Ethics Committee (RA/4/1/5792) and the Western Australian Aboriginal Health Ethics Committee (Ref 476, 1023).

*Study design*: A pilot set of interviews was initially conducted and the questions and interview process reviewed for credibility and confirmability by an advisory board consisting of academics in public health, external community members and an Aboriginal Liaison officer. Questions covered topics such as barriers and enablers towards accessing dental services, culturally appropriate care and how existing dental services could be improved. In this study two female Aboriginal liaison officers and the first author (JP), a non-Aboriginal male researcher and clinician, conducted the fieldwork. The team had existing links with the community and in many instances had already established relationships between participants having worked in clinics or spoken to them over several prior visits to the region. This enabled a degree of familiarity between the researcher and participants during the yarning process. Yarning places value on the participant’s knowledge and expertise and is an open process of sharing between the participant and researcher mitigating the power imbalance that can sometimes occur in conducting research driven interviews [12]. Through this process, participants also shared information that was not directly related to the initial research question but was deemed valuable to their narrative and lived experience. One of the principles of ethical research is that the research outcomes should include specific results that respond to the needs and interests of Indigenous people [13]. As such this data was captured and was considered an integral part of both the contextual understanding of oral health in remote communities as well as being central to the yarning process.

*Sampling and recruitment*: Aboriginal adults over 18 years of age and living in the Kimberley region of Western Australia were invited to participate. Purposive sampling with the assistance of Aboriginal liaison officers enabled invitation of participants leading to a heterogenic sample inclusive of different genders and age groups. Invitations were open, allowing for participation in an individual interview or a yarning group at a time and place convenient for the participant(s). All of those who were invited chose to participate in the study. Each of the interviews was unique with no repeat interviews being conducted, however participants were provided with the contact details of the research team should they wish to add any additional thoughts or concerns. The project was explained and written consent obtained from each participant including permission to audiotape the interview to assist in subsequent transcription. Field memos were also recorded to describe aspects such as the environment and initial concepts arising from the yarning. The final sample size was not formed a priori but rather was dictated by a theoretical saturation and representativeness achieved through reiterative comparison of themes arising from the data [14].

*Data analysis*: Data analysis followed the principles of constructivist grounded theory outlined by Charmaz [15]. Analysis occurred concomitantly with data collection and followed an iterative and cyclical process of gathering data, transcription and coding ultimately leading to data saturation that was attained when no new properties emerged and the same properties continually emerged [15, 16]. Each interview was manually transcribed and initial coding was assisted by NVivo 12 software (QSR International Pty Ltd. Version 12). Additional review of the coding and transcripts were also conducted by LSS and AD. Participants were grouped by gender: AF (female), AM (male) and received a unique numerical ID: AF_1, AF_2, AM_3 etc. Concept maps and diagramming were also used to aid understanding in the construction of categories [15]. The analysis was reviewed by an external committee that included Aboriginal and Kimberley representatives. Furthermore, the research methodology and final manuscript was reviewed by an Aboriginal leader from the Binarri-binyja yarrawoo representing the East Kimberley. The study also followed the National Statement on Ethical Conduct in Human Research, (especially Chapter 4.7 Research with Aboriginal and Torres Strait Islander Peoples) and the Guidelines for Ethical Research in Australian Indigenous Studies [13, 17]. A consolidated criteria for the reporting of qualitative data was also used in presenting the study findings [18].

## Results

A total of 23 individual interviews and 17 yarning groups were conducted which involved a total of 80 participants (27 males and 53 females). The majority of participants were from communities in the East Kimberley including the Fitzroy Valley and the Halls Creek district which covers an area of over 143,000 square kilometres. An estimated 3000 people reside outside the main Halls Creek township in surrounding remote communities extending south into the Great Sandy Dessert. This study includes participants from each of these communities, however to maintain confidentiality the names of individual communities have been de-identified in the transcripts (e.g. Community A).

The results of this study were broadly categorised into enablers and barriers to dental care. Enablers to care included: provision of culturally appropriate care, the use of mobile dental services, heightened awareness of existing services and supplemental services provided by visiting volunteer teams. On the other hand, barriers included limited transport, affordability of services, the complexity of appointment systems, dental anxiety, practitioner experience, hospital-based treatment, shame associated with oral health and health-seeking behaviours and the impact of social determinants on oral health such as housing, poverty and transgenerational trauma.

## Enablers to care

### Mobile dental services

Creating culturally appropriate services relies on oral health professionals to engage with the needs of a community. This level of community engagement is often stifled by the dominance of western education models and the operative approach to dentistry that confines practitioners to conventional clinical settings:

“the dentists, the white people they’re afraid to speak to people that’s why they wait for people to come to the hospital and open the door… you need to go out and search what people need” (AF_41)

The use of a mobile dental clinic in the community was favoured by the majority of participants. Participants felt the need for *“dentists to come out to community”* and felt that the *“mobile van is better”* than the conventional dental clinic. Several reasons were reported from the openness, ‘walk-in’ clinic model to the advertising/promotion created by a large mobile clinic pulling into the centre of town.

“well I think down there you are more open to the public having that there, people can pop in and have a look they have the option of being seen whereas people I don't think like to come to the hospital” (AF_6)

Participants felt that the use of mobile services could potentially increase reach to remote communities acknowledging the transient lifestyle of many living in these regions. Furthermore, the use of mobile clinics may potentially also remove many structural barriers to care such as the need for accommodation and transport to dental services in larger towns:

“Well I reckon if you did have the mobile vehicle…you would cover more ground and a lot more people. It's just the transient people if they're not at (Community A) and they're here well then they all still get in here but the ones from (Community A) that never leave will never be seen” (AF_51)

Moreover, residents in these communities were familiar with mobile clinics being used across other disciplines:

“the mobile clinic would be ideal because they have the mobile clinic for the breast cancer like they've got the truck and things like that, yeah ears but it would be good for teeth” (AM_55)

### School based services

Schools in remote Aboriginal communities provide not only education but also act as a central hub for community health promotion. The school setting was perceived as being an important site for dental service delivery as it creates an equal playing field facilitating access to care for all children:

“every kid at school gets seen, whereas if you set up at the hospital parents might not bring them to get seen” (AF_50)

Many participants recollected the school dental service as being a predictable and memorable experience in their childhood, however they felt that the service had gradually disappeared over recent times:

“yeah we used to like the dentist coming to our school because it meant we having your teeth checked going to the dentist and it's so cool you know just to see what they do and stuff like that but these days it doesn't happen lots has changed” (AF_52)

The transition from school-based services to adult dental services or the lack thereof was cited as one of the reasons behind a decline in oral health:

“the kids have their dentist there at the school…but for the adults yeah it's pretty hard we have to come either here to (Community A) or go into (Community B), I'll have to book an appointment somewhere…” (AM_55)

Similarly parents underlined the value and convenience afforded by a school-based dental service when compared to taking a child to an external dental service:

“taking your daughter out of school getting her to the clinic sitting and waiting, with three or four hours of wages lost…everything does come back to money” (AF_51)

### Awareness of existing services

Participants recognised the availability of a variety of health services in remote communities but the lack of awareness and promotion of these services prevented optimal uptake:

“there are a lot of good services out here but they don't promote them…lots of guys want to go and get their teeth but they don't know how to go about it.” (AM_37)

Many participants commented on the lack of awareness of existing services particularly in the context of itinerant dental services:

“no-one knows if there is a government dentist, everyone finds out two days after. Oh! the dentist was here I didn't even know!” (AF_43)

Services that had pre-arranged posters and promotional material were found to be useful in increasing community awareness. The increasing use of social media among Aboriginal communities has also led to this being a useful platform for health communication:

“I just sort of walked in … they (government services) don’t have much advertising, the (volunteer services) had it on Facebook and around town, whereas I actually had to ring here and find out” (AF_50)

### Volunteer services

Public dental services in the Kimberley are operated by the state-run Dental Health Service (DHS), a means-tested co-payment for treatment is required and clinics currently exist in larger towns in the Kimberley. The DHS also provide outreach services to remote Kimberley communities however the frequency of these visits is highly variable, with the West Kimberley typically being better serviced than the more remote communities in the East Kimberley [19]. As a result volunteer-led services namely KDT, have been employed to ‘fill the gaps’ and extend dental care to these communities. The Kimberley Dental Team (KDT) is a non-for-profit, volunteer organisation established in 2010, the team conducts annual visits to the Kimberley spread over three months each year, with rotating teams of dental volunteers. Following community request and invitation, the KDT provide voluntary treatment at no cost and work in partnership within existing primary health care services or by using mobile dental clinics.

Participants recognised several differences between the volunteer-based model of care and publicly funded dental services. For example, volunteer organisations often bring larger teams to communities and are able to turnover high levels of productivity during their outreach visits:

“only two of them (from the government) really come down whereas KDT have a whole team” (AF_50)

The majority of treatment provided by volunteer services is based on symptomatic care and caters for a combination of walk-in and booked appointments. This approach enables access to a service for acute problems while maintaining the convenience afforded by a scheduled appointment:

“maybe you could have one of them that does do appointments for the working people that never get to come and the other one sit and wait” (AL_51)… “because if you've got toothache and you've got a swollen-up face you want to go straight there, you basically aching” (AL_56)

When speaking about the treatment provided by volunteers, participants emphasised the friendly environment and team-based approach to care:

“(they) come as a team, are very friendly even the setup that (they) have in there when (they) are dealing with us…you've always got someone there” (AF_56)

However, the sustainability of volunteer-based care remains in question with some participants demonstrating a reliance on volunteer-based care given the familiarity with the “*volunteer mob*” and actively seeking out free services:

“well for me I haven't been to a dental service, I’ve always waited for the volunteer mob, I never go to this other mob I don't really know them” (AF_56)…“yeah they won't pay, they'll wait for the free dentist” (AF_46)

When comparing the volunteer-based services with publicly funded services, participants recognised that volunteers provided a stopgap measure and that a more predictable long-term solution was needed. The uncertainty around when services are available has been underlined by similar fly-in fly-out models of care [20]:

“it's good that volunteers come down but we need someone based here regular because when someone has tooth problems they have to wait weeks on end to get seen so sometimes the communication is not good, no one knows that you are in town whereas if they know that you are based here they know that they can get their tooth checked or tooth pulled out” (AF_4)

## Barriers to care

### Transport and affordability of services

Lack of transport to and from the nearest dental clinic was an overwhelming barrier to care, reported by almost all participants:

“Being in (Community A) in the remote area, dentists are only based in (Community B) and (Community C) so we'll travel out and yeah it's difficult for families and for anyone to go to the hospital.” (AM_37)

Factors such as looking after children, leaving family behind, finding accommodation while accounting for both travelling to a service and finding a way back home all impacted on participants ability to seek care:

“people just can't get in, they can when they have transport, they can get dropped off but they don't know if they can get back” (AM_18)

Furthermore, travelling away from community was also associated with a fear of the unknown and resistance to seek care:

“(Community A) is an isolated township it's four hours that way and seven hours to (Community B)…we have outlying communities and we have a lot of Aboriginal people that don't have transport and they're too frightened to go up there” (AF_21)

Additionally, the lack of publicly funded dental services was noted by participants who also remarked on the disconnect between public medical care and dental care:

“I've been to the one at the hospital to get my tooth pulled out the cost was $300 and you know that’s too much and people can’t afford that, you've got Medicare but it doesn’t cover that....it's very hard living in the Kimberley and having your tooth taken out” (AF_56)

### Appointment scheduling

The need to schedule appointments and complete preparatory paperwork presented additional logistical hurdles when accessing public dental services. Stories were recounted where people had arranged their travel and accommodation to seek care at their nearest service only to be turned away because of incomplete documentation:

“you have to bring your Medicare cards and things like that they need to know what to bring …turning up there in agony and coming to see somebody, you get turned away because you have to bring a card you have to get all the stuff” (AM_24)

The ability to have a walk-in dental service, may remove many of these barriers providing those in remote communities with access to emergency dental care*.* However, an emergency service such as that offered in a tertiary hospital often results in excessively long wait periods and this was a source of frustration for many:

“a lot of people don't like going to the hospital because just like what I said if you're sitting there for two hours and I'll be waiting for the doctor to give you one Panadol, Jesus Christ I could have walked down to the shop” (AM_27)

The concept of time is fluid in remote communities and appointment structures are largely foreign and only used in specialised settings such as in the hospital system:

“people don’t know about appointments it’s something new to most of us Aboriginal people…only time you use appointments is when you going to the hospital or doctor but they don’t really know about that anyway” (AF_56)

### Pain as a motivator for care

Pain was an overwhelming motivator to seek care and was the most common reason influencing participants in their decision to seek dental care:

“a lot of people don't clean their teeth you don't see them going into the shop and getting toothpaste and toothbrushes they just wait and wait and wait until something happens…they look for the toothbrush and toothpaste when they are in pain, that's the only time” (AF_43)

The self-perpetuating cycle of symptomatic care results in band-aid solutions to dental care and contributes to the prevailing fatalistic attitudes towards oral health. As discussed by one participant who attended a dental clinic with a toothache:

“well I'm not sure whether the options were there because you're straight in sort of like emergency fix it” (AF_51)

On the other hand, many parents, who had already experienced dental pain in the past and had required emergency treatment showed a proactive approach towards preventing dental disease for their children:

“they don't think that they can overcome that problem and even I say to my kids if you don't go to the doctor how do you expect to get better” (AF_6)

“I don't want to see my kids with no teeth, that's why I get toothbrush and toothpaste for my kids to keep their teeth clean”(AF_46)

### Dental fear

One of the commonly reported barriers towards seeking dental care was dental fear and anxiety and the idea that *“the dentist will probably hurt them”*. In many instances this was associated with previous negative experiences at the dentist:

“if you see a dentist, I'd go the other way (laughing)… they use to be grumpy in my day” (AM_17)

While in others it was a preconception of fear:

“I didn't want to go up there I wasn't game enough” (AM_32)

Additionally, as care is often symptomatically driven in remote communities, the delay in accessing timely care and suspected treatment propagated further dental anxiety. This was particularly true in the case of extractions and the thoughts around the surgical procedure involved:

“probably scared some of them probably think that their teeth are too far gone” (AM_3)

“the needle and the way they pull it out with pliers” (AF_43) … “its afraid for them pulling it out, nervous you know, that’s the only thing” (AF_43)

### Hospital-based dental clinics

Most remote communities in the Kimberley are serviced by itinerant dental services. For the most part dental clinics in these communities are integrated within the main hospital. Although this affords the dental team the ability to work within a multidisciplinary setting with easier access to wider health infrastructure, it also brings with it the stigma associated with the hospital environment:

“the hospital can be a scary place at the best of times… bad experiences and you know Aboriginal people die in that building that type of thing” (AM_27)

“Hospital is the last place for people to go…they only go when they're sick and for bad news” (AM_37)

Despite these issues, the hospital was often the primary source of care for many in community and when asked what you would do if you had a toothache “*ring the hospital*” was a recurring response. However, the majority of participants conveyed a sense of helplessness when seeking dental advice from primary health care providers and reinforced the lack of service availability:

“they just give you Panadol and send you home but it doesn’t work” (AM_49) or “they won't do anything they will just tell you to wait for the dentist” (AF_48)

Participants also recognised that seeking this sort of symptomatic care was a stop-gap measure and placed an additional burden on the health system:

“every minute go to the nurse, humbugging you know, in paining like that, pain, pain, have that pain all the time you know” (AF_47)

### Shame

In talking about the hospital environment, the notion of shame was often reiterated and participants discussed the connection between shame and the hospital:

“Like the younger guys under 30 when you go to hospital people judge you know you feel people looking at you, they look at you as not healthy…people jump to conclusion these days there's a lot of bad rumours flying around from the medical service area.. Bad history you know” (AM_37)

Open communication and approachability was deemed to be key towards overcoming issues such as shame, as noted by one of the participants:

“communication and understanding you know of what is around, I'm part of a team, we have a men's circle every Wednesdays and we encourage fellas to lift their head up and if they have anything to talk through and if they have anything in their personal life everything is confidential” (AM_24)

The concept of shame also raised some tension between an individual’s willingness to seek care and the accessibility of health services:

“Culturally I think being shamed is the biggest excuse sometimes…Well me, myself I don't want to come to the dentist because I'm scared and I'm not in pain yet but the day that the tolerance hits I'll be the first one at the door. So it's not that I'm shamed it's half that I'm not in really severe pain and I'm just going to make every excuse under the sun to blame someone else for it” (AF_51)

## Impact of social determinants of health

The willingness for members of remote Aboriginal communities to engage and openly communicate with health services may be influenced by the impact of cultural oppression and the intergenerational consequences of the legacy of colonisation, racism and discrimination:

“I would say here in (Community A) it's a very small community and most of the population is Aboriginal so when they go to a big city they realise that they are the minority they are not like white people…I don't know I think maybe because of the European history they came in and took over leaving Indigenous people as the minority their views don't matter…look at how we've lost our culture we've lost our language” (AF_6)

These discussions, however, raised questions around perceived and true barriers to care while also raising the question of whether these were due to systemic societal inadequacies or whether they were based on other factors such as fear, lack of knowledge or confidence. Between participants there was a sense of frustration and a divide in attitudes as to why some people are motivated to seek care while others are not:

“the services are available, they are really really good but people have to ask for help or actually go to the dentist to be seen to get all the stuff, you can't just sit at home and say my child's got bad teeth give them coke and then not go to the dentist, some people actually want to be picked up by the dentist and their hand held” (AF_51)

## Practitioner preference

Several strategies have been used to encourage clinicians and new graduates to rural and remote areas [21, 22]. However, participants were cautious about inexperienced practitioners training in communities and valued the care provided by experienced clinicians:

“you get other dentists going there who are not even really trained or professionals they're just going there to get their marks for uni and all these kids will be guinea pigs they're not really getting professionals or top dentists” (AM_5)

Previous traumatic dental experiences were often vividly recounted by participants and these negative experiences have the potential to propagate further dental anxiety in remote communities:

“I think he was a terrorist, well he pulled it out and broke the tooth getting it out…. I’d need to be in alot of pain to go back” (AM_58)

When asked about practitioner preference most participants felt that they preferred *“the most experienced, you want the best, someone who knows what they're doing” (AM_49)*. Contrary to popular belief seeing a dentist with an Aboriginal background was not perceived as being an important factor and in fact participants in this study indicated a preference for a dentist to be external to the community:

“I don't think blackfellas trust one another (AM_44)…Aboriginal to Aboriginal don't trust one another from here (AF_44)…especially when we lived here, it’s alright from another place, a stranger you know, that we don’t really know them very well, so that’s alright (AF_44)… if they put me as a dentist no one will come to me, they'll trust you but not me” (AM_44)

There was a strong push for a predictable service in the first instance rather than any particular preference on who would be running the service:

“we don't have a preference anybody can look at our teeth, our teeth are important, we don't care if a man take them out or do fillings or whether it be a woman or whether its Aboriginal, non-Aboriginal as long as they fix us up” (AF_56)

Nevertheless, the role of gender was acknowledged as being important particularly when treating elders in the community:

“well I suppose back in the old days, some men wouldn’t like women treating and some women wouldn’t like a man” (AF_50)

Interestingly one of the participants commented on the impact of cultural oppression over time and the value of health practitioners being active listeners rather than being prescriptive or dogmatic:

“I feel more comfortable with people of colour, they know, they know how to talk to them (Aboriginal people) and give help to them rather than the white person who comes down and says you need to do this this and this or whatever” (AF_6)

## Discussion

Participants in this study indicated that oral health and access to timely dental care is important to Aboriginal people living in remote communities, however current service delivery systems are struggling to address the levels of acute need. Transport to and from the nearest dental service, accommodation and the costs associated with dental treatment have all been underlined as barriers to care in the literature and further reinforced by the findings of this study [5, 6, 20, 23]. Although participants expressed the need for a predictable, on-site dental service, the low population densities combined with the high running costs of a dental practice means that establishing a permanent dental workforce and full-time services is generally not viable nor sustainable in many remote communities [24, 25]. This is similar to the barriers faced by other remote communities across Australia where workforce challenges and funding allow for only intermittent dental services supplemented by models such as a fly-in-fly-out service [20]. Nevertheless, this study highlighted that the services to remote Kimberley communities, albeit itinerant, often go unnoticed and that heightened community awareness, advertisement and publicity is required to facilitate their uptake.

The impact of mobile dental clinics in remote Aboriginal communities is an area that has not been significantly explored in the literature. The basic function of these mobile units is to serve as ancillary services to the operations of the recipient hospitals especially in priority, underserved, hard to reach and remote areas [26]. Mobile clinics confer several advantages ranging from the visual publicity and awareness of a health service through to the ability to setup in an open outdoor space which participants perceived as being more welcoming and approachable. This form of service delivery is commonplace in remote Aboriginal communities with programs such as BreastScreen WA's mobile service and the Earbus program [27]. A multidisciplinary, integrated visiting service model based on common risk factors could therefore be used to increase the impact of health promotion and service delivery to remote Aboriginal communities as reflected by the question posed by a community member *“does this dental mob do eyes too?”*.

Community consultation and ‘yarning’ was found to be strongly valued in breaking down barriers and better informing the services required by a community [28]. The use of mobile clinics in remote communities may both extend the reach of services and have the potential to facilitate a deeper level of community engagement by encouraging practitioners to break free from the confines of the conventional four-walled dental clinic.

Alternative strategies to complement existing public dental services have been recommended in the Kimberley region [29]. One of these is the use of dental volunteers such as the KDT, to extend dental care and oral health promotion activities to remote underserved communities. Participants recognised the variances between a volunteer-led model and public dental services primarily citing the larger team-based approach, use of mobile dental clinics to extend reach to outlying communities and a combination of walk-in and scheduled appointments which were all perceived as being enablers to dental care. However, this study raised several limitations around relying on volunteer based care including sustainability of services, the potential for volunteer services to devalue the care provided by mainstream services and the persisting gap in care that remains once itinerant services leave [20, 30]. The role of volunteer groups may therefore be more appropriate in supporting local preventive programs, building oral health capacity and increasing oral health advocacy. For example, the school-based toothbrushing program supported by KDT was viewed favourably by participants in this study who suggested that these early interventions may facilitate positive oral health behaviours in children as they grow.

Social and oral health-related indicators whether they be DMFT, literacy or socio-economic status are typically lower for the Indigenous population when compared to the non-Indigenous population [23]. However, these statistical truths are insufficient in ascertaining the self-perceptions, lived reality and sociocultural practices of Aboriginal people. Multidimensional concepts such as shame, for example, are difficult to measure, describe and are contextually fluid yet play a significant role in an individual’s willingness and ability to seek care as shown in the results of this study. Shame has been described as a social emotion that originates in response to a threat to a social bond with the subsequent fear generating social conformity [31]. Additionally, shame may be more pervasive in communities where it is compounded by existing inequalities. The concept of shame reflects a state of low self-esteem and has been related to discrimination, marginalisation and poverty, when combined with social and environmental stressors, trauma and disempowerment this creates a significant barrier to optimal oral health and health seeking behaviours [31]. A neoliberal discourse suggesting that individuals should be held responsible for their own health was raised in discussions around shame. This included choices such as buying a toothbrush, going to the dentist or sending their children to school. However, whether these choices truly exist is debatably a product of a number of psychosocial factors and inequalities such as income, education and socio-economic status. Notably the participants that spoke about the importance of responsibility and recognised the consequences of their actions tended to be in positions of power in the community, had stable jobs and were aware of the intricacies of the health system at large. The emotional, physical and financial impacts of oral health are not unique to remote communities. These domains along with aspects such as competing health priorities, costs of care and mistrust of the health system are similar to those reported among Aboriginal families living in urban centres [6, 32]. This further consolidates the evidence underlining the pervasive structural inequalities impacting on Aboriginal oral health [32, 33].

The yarning circles conducted in this study revealed that the gender or Indigenous status of a practitioner were not barriers to care and in fact participants preferred the dentist to be someone from outside of the community to mitigate feelings of shame or familiarity. This is in contrast to the preference for Aboriginal practitioners or gender specific health services seen in primary health care especially when dealing with sensitive health issues such as sexual and mental health [34]. Notably issues around trust and distrust of practitioners and the health system were raised in this study which echo the historic experiences of racism, mistreatment and discrimination experienced by Aboriginal people [35]. Gaining and maintaining trust takes time and this is an important aspect in building self-efficacy [35]. However, this is not naturally incorporated into operative models of dental care or dental curricula where there has traditionally been a heavy focus on treating the tooth rather than the person [36]. Previous literature also shows that practitioners with more experience tend to work in urban locations and new graduates tend to start their careers in rural practices [37]. Although attracting graduates to rural and remote practice may address shortages in the dental workforce, young clinicians often have lower levels of cultural competence and are still developing their surgical skillset which may contribute towards feelings of mistrust as expressed by some participants in this study [36]. As a result, building rapport and ensuring relational continuity is critical and clinical yarning may provide an avenue through which future services to gain trust, facilitate shared decision-making and improve self-efficacy among Aboriginal people.

The long-term impact of colonisation and transgenerational trauma are aspects that have, and continue to, generate distrust and fear of health systems within Aboriginal communities [6, 35]. This study echoed the findings of other studies which have highlighted the stigma experienced by Aboriginal Australians in accessing health services such as visiting a tertiary hospital or dental clinic accompanied by the fear of the unknown and underlying dental anxiety [38, 39]. As such the need to deliver culturally appropriate and more importantly culturally secure care becomes critical [40]. Existing dental services rely on Aboriginal patients to conform to the expectations and protocols of the health service. The findings of this study suggest that dental services need to be more inclusive, collaborative and flexible in responding to the needs of Aboriginal people in remote communities and this is more likely to empower and build trust between the service and the community. Rather than the traditional prescriptive and dogmatic approach to dental treatment which is often closely tied with an operative model of dental care, participants in this study appreciated open two-way communication and a friendly, welcoming service where their views were heard and acknowledged. There is also a significant lack of Aboriginal health care workers and liaison officers in the oral health space and incorporating Aboriginal health workers as well as local champions as a first point-of-contact may assist in mitigating fear, unravelling issues such as shame while enabling a more culturally secure service [35, 40, 41].

## Strengths and limitations

This study attempted to capture the views of a diverse range of Aboriginal Australians living in remote Kimberley communities using a qualitative framework. Although, the interviews and yarning circles were conducted with the assistance of Aboriginal liaison officers, the nature of qualitative research brings with it both researcher and participant bias. The primary interviewer, JP, is both a researcher, a clinician and volunteer with the KDT having worked with remote Kimberley communities over the last decade. Traditionally, the need to be an “outsider” in order to objectively assess data has been emphasised [42]. This is to avoid potential bias and maintain authenticity as a researcher’s position may become clouded by their personal experience and membership within a certain group [43]. On the other hand, being an “insider” often allows the researcher a level of trust and openness allowing for more rapid and complete acceptance by their participants and a greater depth of data to be collected [43]. Therefore, the need to be both associated with the emic and etic is essential towards developing and fostering ongoing relationships in remote communities. In order to mitigate the influence of social desirability bias, questions were curated and enhanced through pilot interviews, the data was triangulated by the other investigators who were considered etic to the data. Moreover, theoretical saturation, rather than an ad hoc sample size, dictated the end-point of data collection. Social constructs and variables such as age, gender, ethnicity, socioeconomic status and education can all impact on qualitative research, despite the researchers best efforts at maintaining neutrality [44]. The constructivist theoretical model that underpinned data collection and analysis acknowledges that meaning and knowledge exchange is cofacilitated between the dialogue between participant and researcher [45]. Throughout this process there is a continuous unfolding of perspective that is coloured by the social mileu, background and experiences of those involved. As a result, the results of this study are constrained by the impact that these social determinants may have on certain themes being raised. However, throughout the research process, cultural safety was closely considered and creating a safe space enabled participants to discuss complex and challenging themes and where appropriate these were guided by the female liaison officers rather than JP as a male researcher. The open structure of a yarning circle also yields a large quantity of data that often extended beyond discussions on oral health, within the constraints and scope of this manuscript and a lot of this data could not be included and thematic analysis primarily guided what data was included and explored in depth.

## Conclusions

This study explored the barriers and enablers to oral health care and service delivery in remote Aboriginal communities in the Kimberley. The findings of this study suggest that the impact of cultural oppression and the intergenerational consequences of colonisation, persistent racism and discrimination continue to play a significant role in the disparities in oral health experienced by Aboriginal Australians in remote communities. Furthermore, multidimensional issues such as shame influence not only an individual’s well-being but also their health-seeking behaviours and lifestyle choices. Existing dental services continue to be based on traditional operative models of dental care and rely on prescriptive oral health messaging which may not adequately address the existing structural barriers to care or enable a culturally secure service. Although volunteer-based models of care have been welcomed in extending care to remote Kimberley communities, the sustainability of these services remains a question. The role of volunteers may therefore be more appropriate in supporting local preventive programs, building oral health capacity and increasing oral health advocacy while increased efforts are needed to ensure more predictable and frequent public dental services to remote communities. Integration with existing primary health services and community hubs such as the school, extending the reach of dental services through the use of mobile units and incorporating clinical yarning into dental care are echoed by the voices of community members as showing strong potential in improving the current state of dental services to communities in the Kimberley.

## Data Availability

Data sharing is not applicable to this article as no datasets were generated or analysed during the current study.

## Abbreviations

KDT: Kimberley Dental Team
AF: Aboriginal Female
AM: Aboriginal Male
